# Highlights of the 1st Ecuadorian-Venezuelan Symposium of Young Researchers in Bioinformatics (1SEVJIB)

**DOI:** 10.12688/f1000research.125381.1

**Published:** 2022-09-22

**Authors:** Sebastian Ayala-Ruano, Fernando Hernandez, Arantxa Ortega, Deliana Infante, Daniela Carrascal, Karen Sánchez-Luquez, Rafael Puche-Quiñonez

**Affiliations:** 1 Grupo de Medicina Molecular y Traslacional (MeM&T), Universidad San Francisco de Quito, Quito, Ecuador; 2 Centro de Medicina Experimental, Instituto Venezolano de Investigaciones Científicas (IVIC), Caracas, Venezuela; 3 Grupo Modelado Inteligente de Sistemas, Universidad de Cádiz, Algeciras, Spain; 4 Unidad de Estudios Genéticos y Forenses, Instituto Venezolano de Investigaciones Científicas (IVIC), Caracas, Venezuela

**Keywords:** Bioinformatics; Regional Student Groups; ISCB; Student Council; Virtual symposium; collaboration; networking.

## Abstract

The COVID-19 pandemic profoundly changed how scientific conferences are

organized, fostering virtual meetings. These online events have allowed students

and researchers to overcome geographical, administrative and economic barriers to

attend and organize high-quality international symposiums. Moreover, these virtual

conferences have contributed to the creation of inclusive activities that strengthen

scientific communities. Here, we summarize the main activities and learnings from

the 1st Ecuadorian-Venezuelan Symposium of Young Bioinformatics Researchers

(1SEVJIB), organized by the Ecuadorian and Venezuelan ISCB-SC Regional

Student Groups (RSGs). This symposium aimed to provide an opportunity for

undergraduate and postgraduate students from Ecuador, Venezuela, and other Latin

American countries to share their Bioinformatics research. The 1SEVJIB was the first

bi-national conference organized by two RSGs from Latin America (LatAm). This

symposium was a two-day virtual meeting with five activities: 1) oral student

presentations, 2) poster session, 3) keynote lectures, 4) workshop, and 5) round

table. This conference promoted the scientific exchange and cooperation networks

between young Bioinformatics researchers and students from Ecuador, Venezuela,

and LatAm, giving them opportunities to boost their scientific careers.

## Introduction

Bioinformatics has been among the fastest-growing scientific fields in the last 25 years in Latin America (LatAm), which is reflected in the growing number of publications in the area across countries that constitute this region. However, Brazil, Mexico, Argentina, Chile, and Colombia are the countries that concentrate around 98% of the Bioinformatics articles in the period between 1991 and 2016. In the same time span, there were only 32 Bioinformatics articles from Ecuador and Venezuela, representing a very low percentage compared to other countries in the region. ^1^ This data indicates that there are necessary steps that need to be taken in order to improve Bioinformatics research in Ecuador and Venezuela; considering that both countries have human resources actively working in research related to the analysis of massive biological data.

Regional Student Groups (RSGs) of the International Society for Computational Biology Student Council (ISCB-SC) are initiatives that could contribute to the development of Bioinformatics, connecting students interested in this field from all over the world, and creating more awareness about Bioinformatics education. ^2^ For example, the foundation of RSG Chile in 2015 was a key step to promote the creation of the Chilean Society in Bioinformatics that happened several years later in 2021.

LatAm joined this initiative of Regional Students Groups in 2012 and there are currently nine RSGs (Argentina, Brazil, Chile, Colombia, Costa Rica, Ecuador, Mexico, Peru, and Venezuela) with Ecuador and Venezuela being the most recently incorporated groups. In this way, Ecuador and Venezuela were placed in a global community working to enhance Bioinformatics locally and globally.

During the recent years, different collective actions have been articulated to potentiate Bioinformatics in the LatAm region through the organization of symposiums. ^3^^–^^6^ However, isolated efforts in Ecuador and Venezuela have been insufficient to contribute to this endeavor. Thus, these countries have not had an academic space where students and professionals can share their advances in Bioinformatics.

In this scenario, we organized the 1st Ecuadorian-Venezuelan Symposium of Young Bioinformatics Researchers (1SEVJIB), the first bi-national conference organized by RSGs of LatAm. In this event, we prioritized the needs of students from Ecuador and Venezuela, as well as from other LatAm countries. Therefore, this symposium represented a new opportunity for students from different LatAm countries to connect and participate in various aspects of Bioinformatics research. It is important to consider that this symposium was the first national and bi-national activity that focuses on Bioinformatics and Computational Biology research in Ecuador and Venezuela. The event was conducted in Spanish.

## Scope and format of the symposium

This event aimed to provide an opportunity for undergraduate and postgraduate students from Ecuador, Venezuela, and other countries from LatAm to share their Bioinformatics research, and create a broader community in the region of early career researchers in this field. Due to the COVID-19 pandemic, we organized this conference as a virtual and free of charge edition, following the guidelines adopted by the ISCB-SC since 2020. ^3^^,^^6^^–^^9^

**Figure 1.**
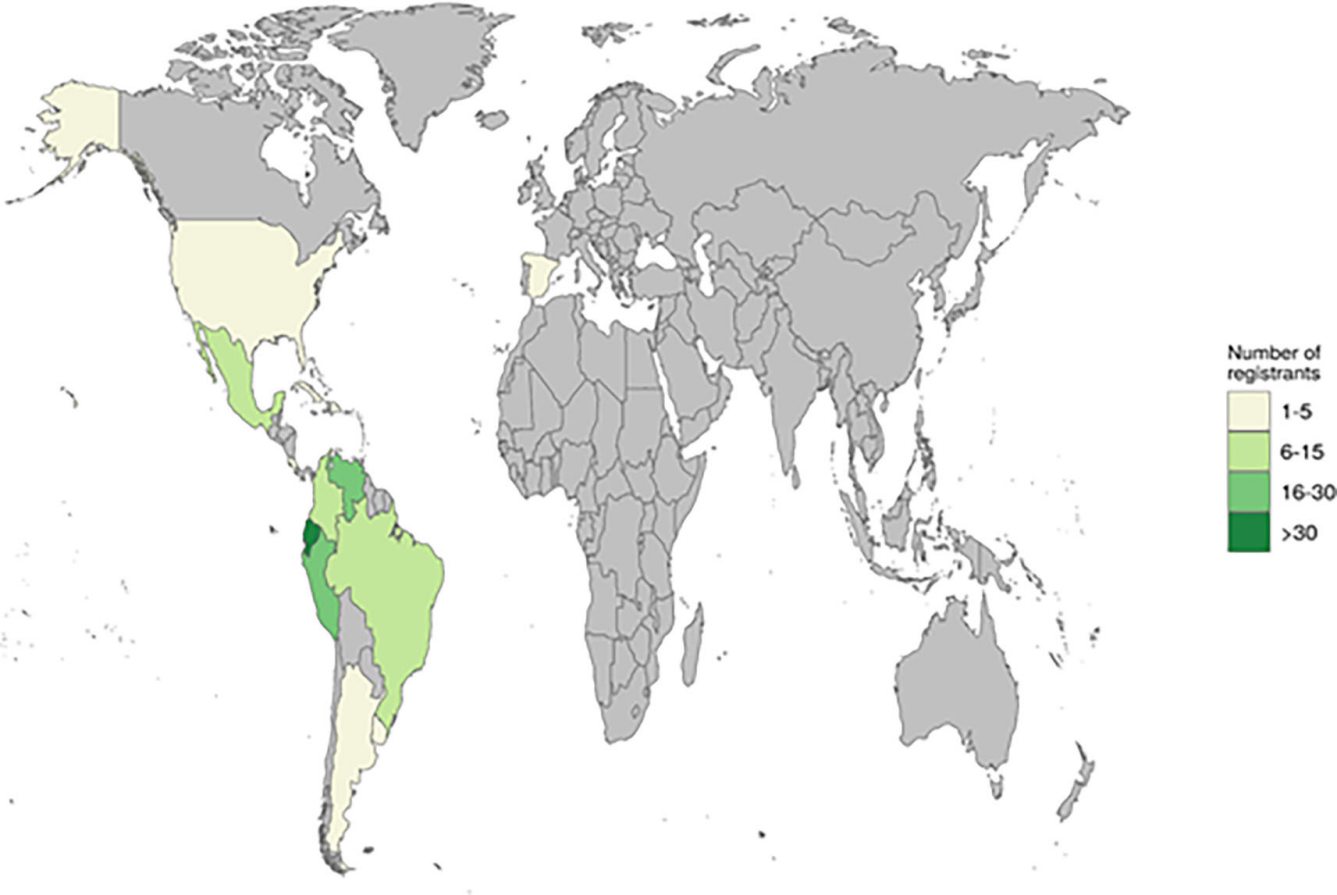
World map of registration to the 1SEVJIB. This figure was created with the ggplot2 R package. ^10^

The format of the 1SEVJIB was a two-day virtual meeting with five activities: oral presentations of students, a poster session, keynote lectures, a workshop, and a roundtable. On the first day of the symposium, we had the oral presentations, poster session, and keynotes lectures; while the workshop and roundtable took place on the second day. We used the Zoom and AirMeet platforms for this online symposium. The recording of all symposium’s activities is available on the RSG Ecuador Youtube channel in the playlist: “ Primer Simposio Ecuatoriano-Venezolano de Jóvenes Investigadores en Bioinformática”. The event had 172 registered participants from different countries in LatAm, USA, and Europe, as is depicted in Figure 1. As we expected, we had more participants from Ecuador and Venezuela, but the virtual format allowed the attendance of students from other countries as well.

Our keynote speakers were three renowned LatAm researchers: Lucía Spangerberg (Institut Pasteur de Montevideo, Uruguay), Paúl Cárdenas (Universidad San Francisco de Quito, Ecuador), and Alejandra Medina-Rivera (Universidad Nacional Autónoma de México, México). In addition, we offered a workshop titled “ *Immunoinformatics as a tool to combat the current SARS-CoV-2 pandemic”*, guided by José Marchan (Karolinska Institutet, Sweden). To close the event, we organized a roundtable discussion about the Bioinformatics situation in LatAm with four invited panelists: Ascanio Rojas (CeCalcULA, Venezuela), Hugo Naya (Institut Pasteur de Montevideo, Uruguay), Francisco Flores (ESPE, Ecuador) and Vinicius Maracaja-Coutinho (Universidad de Chile, Chile).

## Oral and poster presentations

We received 15 students’ proposals that were evaluated by five independent reviewers. Then, six abstracts were selected for oral presentations and eight for poster presentations ( Table 1). The speakers were students from different academic levels, including Ph.D., master's, and undergraduate students from various LatAm countries. These presentations comprised a wide variety of Bioinformatics topics, including genomics, structural bioinformatics, phylogenetics, among others. The oral presentations were 15-minute pre-recorded talks transmitted during the main session of the symposium, where students explained their projects and answered questions from attendees at the end of their talks. The poster presentations were held as short talks hosted during a 1-hour poster session.

**Table 1.** Oral and poster presentations summary.

Title	Presenter	Presentation type
Alterations in the expression profile of miRNAs in colorectal cancer patients	Anthony Vladimir Campos Segura	Oral presentation
Cloning and in silico analysis of the coding region of the interleukin-17 gene of *Argopecten purpuratus*	Yurubí Amaru Borregales Reveron	Oral presentation
Effect of homologous compounds present in ivermectin on the structural stability of proteins associated with COVID-19	María Laura Hurtado-León	Oral presentation
Boolean logic modeling of macrophage polarization	Viviam Solangeli Bermúdez Paiva	Oral presentation
Bioinformatics analysis of tridimensional antibodies structures associated to SARS-CoV-2	Lady Belén Maldonado Cuascota	Oral presentation
Design of a fusion protein to be expressed in microalgae as a vaccine against the Andes virus: a computational approach	Daniel Garza	Oral presentation
Importance of Big Data in Marine Science. Usage of the Whatsapp platform on forums-chats modality as a method of diffusion in scientific topics, related to careers: Marine Biology, Aquaculture, and Food Technology.	Jesús Manuel Villarroel Rojas	Poster presentation
Design of a fusion protein for expression in tomato as a vaccine against SARS-CoV-2: a computational approach.	Daniel Garza	Poster presentation
Anemia in Ecuadorians women of reproductive age: data from the national survey - ENSANUT 2012.	Sharon Antonella Reinoso González	Poster presentation
Usage of digital applications for the dissemination and knowledge of marine macroalgae.	Nelson Ramirez	Poster presentation
Stability of protein-ligand complexes between glucosyltransferase and citronellal terpenes.	Mario Andrés Jurado Herrera	Poster presentation
The use of remote sensing as a tool to determine changes in mangrove forests.	Mariana Rondon	Poster presentation
Discovery of antimicrobial peptides in spiders silk glands using *Expressed Sequence Tag Data.*	Alex Fabricio Sánchez Yumbo	Poster presentation
P(d)PANA: a vaccine candidate against COVID-19 made by young Venezuelan scientists.	Javier David Uzcátegui González	Poster presentation

## Keynote speakers

**Figure 2.**
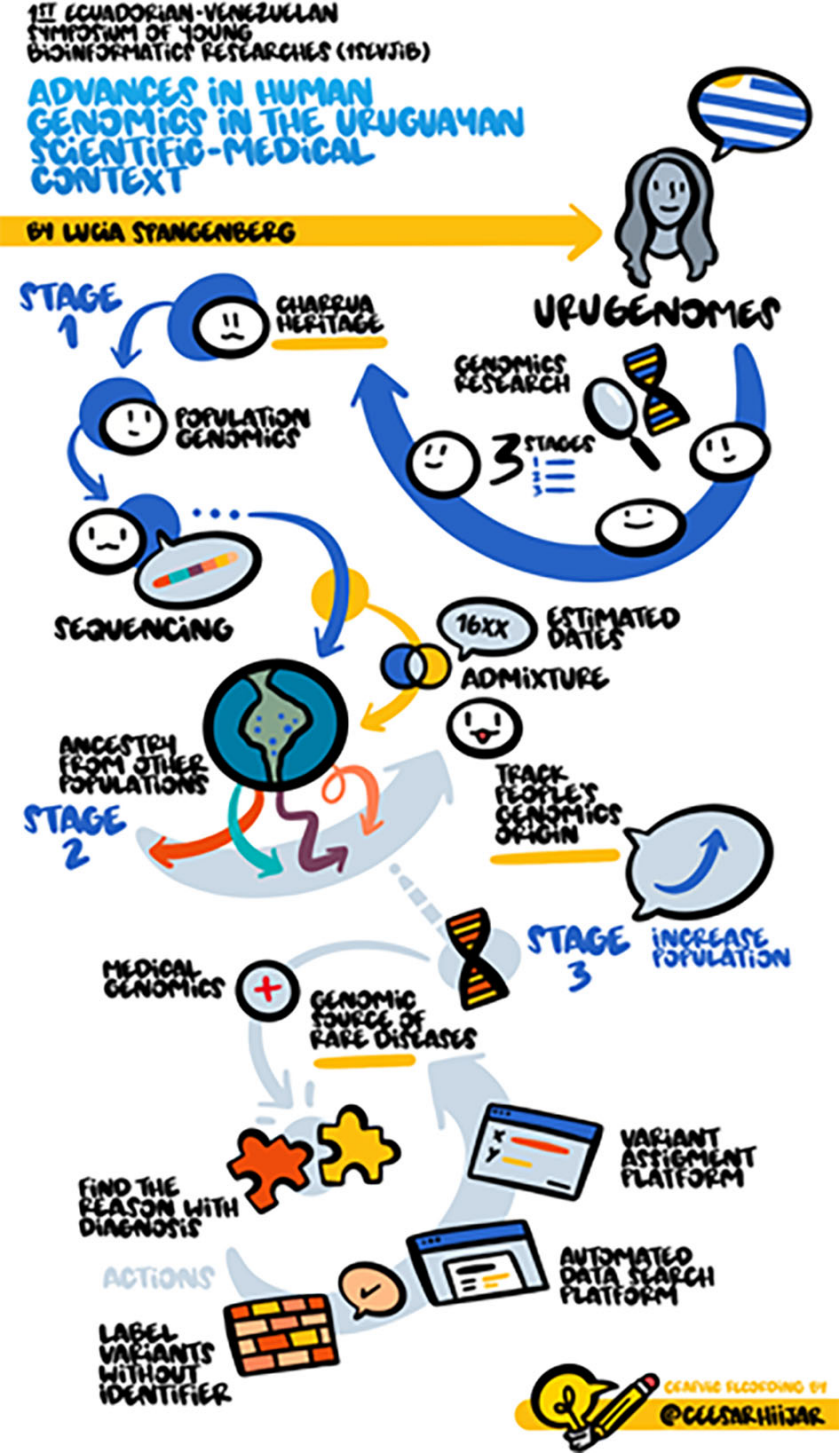
Graphical summary of the keynote presentation of Dr. Lucia Spangerberg. © Cesar Hijar 2021.

The first keynote speaker was Dr. Lucia Spangerberg from the Bioinformatics Unit of Institut Pasteur de Montevideo. She divided her talk into 3 parts. The first part commented on the advances that Uruguay has made in population genomics and the ancestral trace in the Montevideo population. This project is yet to include a larger number of people, but there were already preliminary results that revealed a 14% autochthonous indigenous component in Uruguay’s population. In the second part of her talk, she commented on these results in a clinical context, also how her research group with different sequencing approaches has found the diagnosis, and in most of the cases, these findings contributed to the improvement of the quality of life of these patients. Finally, and rounding up the two previous examples, she comments on the development of Machine Learning tools, ^11^ which are intended to support the process of recognition and classification of missing data imputation on features associated with genomic data. A graphical summary in English of this presentation is available in Figure 2.

**Figure 3.**
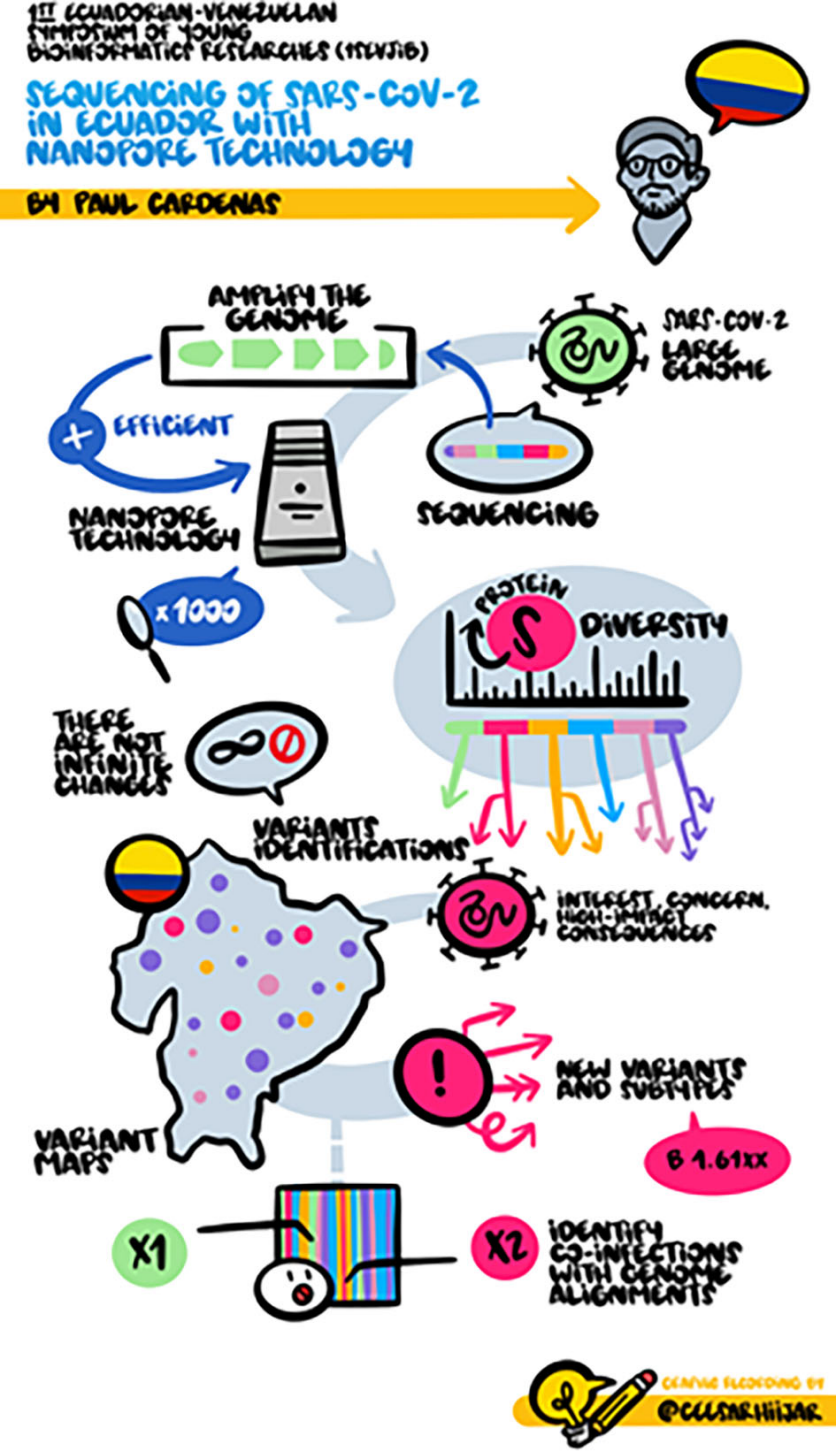
Graphical summary of the keynote presentation of Dr. Paul Cardenas. © Cesar Hijar 2021.

The second keynote speaker was Dr. Paúl Cárdenas, who is head of the Bioinformatics Center at the Universidad San Francisco de Quito. He mentioned Ecuador's track record in sequencing SARS-CoV-2 during the current pandemic. ^12^ Moreover, he commented on the protocol held for this purpose and how the implementation of bioinformatics tools has benefited the recognition of new variants and the temporal appearance dynamics of these variants in different cities of Ecuador. Dr. Cardenas also presented a detailed description of SARS-CoV-2 genomic epidemiology in Ecuador, ^13^ and how all this surveillance has been developed with the high-quality and low-cost sequencing technology MinION (Oxford Nanopore Technologies). He mentioned the importance of these technologies for countries like Ecuador, which have low financial resources for developing epidemiological genomic surveillance. Figure 3 shows the graphical summary of this presentation.

**Figure 4.**
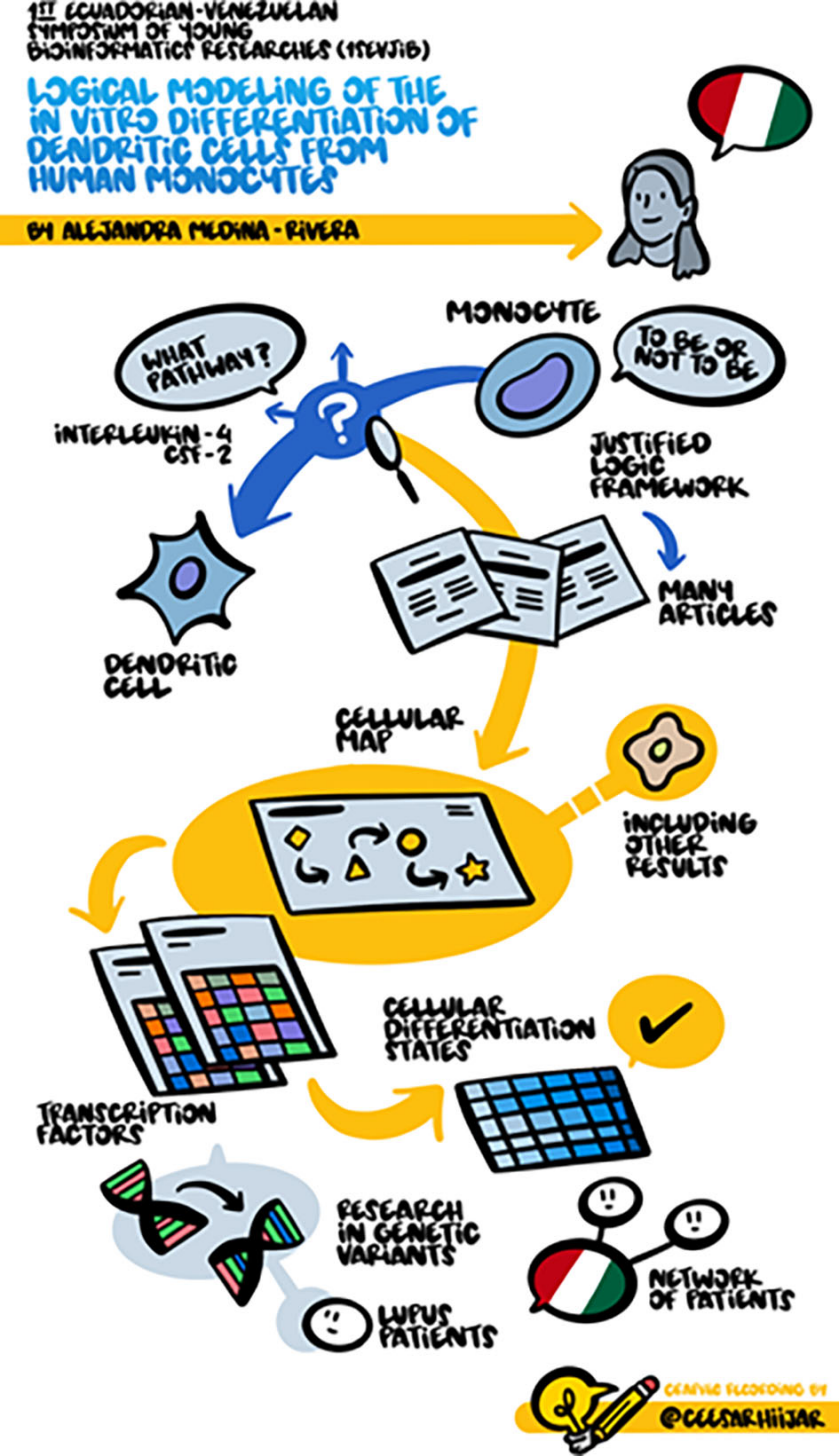
Graphical summary of the keynote presentation of Dr. Alejandra Medina-Rivera. © Cesar Hijar 2021.

The third keynote speaker was Dr. Alejandra Medina-Rivera, who is head of the Regulatory Genomics and Bioinformatics Laboratory at the National Autonomous University of Mexico. She presented how computational modeling allows further knowledge of cell signaling pathways, expression prediction preferences, and cell differentiation. Dr. Medina-Rivera explained that by simulating the results of *in vitro* experiments, computational modeling has allowed remarkable advances in the understanding of human monocytes' differentiation into dendritic cells. She mentioned that this research unraveled novel transcriptional regulatory interactions, which would be particularly useful to understand immunological disorders at the clinical level. ^14^ The graphical summary of this presentation is available in Figure 4.

## Round table discussion

**Figure 5.**
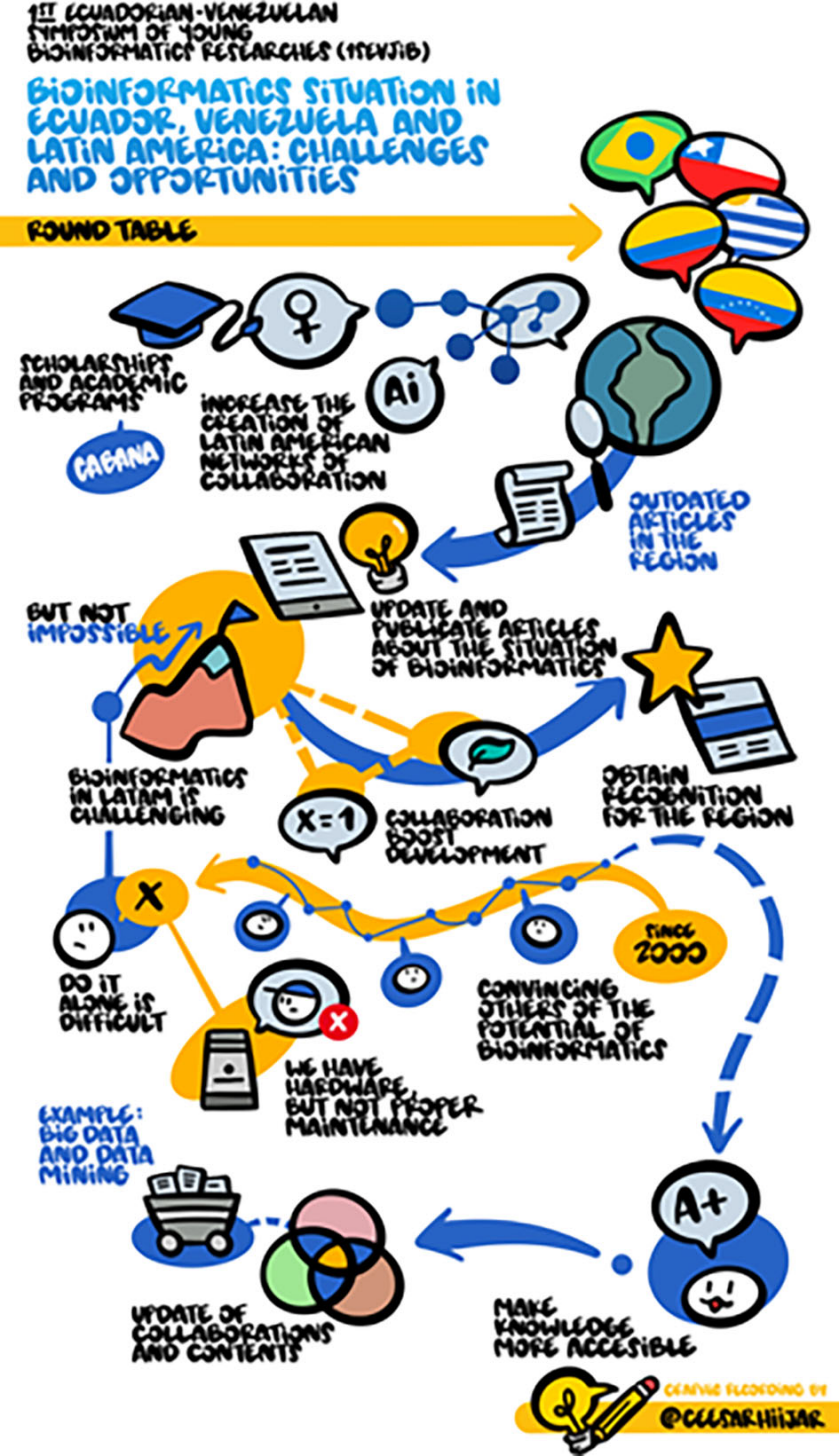
Graphical summary of the round table discussion. © Cesar Hijar 2021.

At the round table, each of the speakers talked about the situation of Bioinformatics in their countries and LatAm. Dr. Vinicius Maracaja-Coutinho explained a global perspective of the situation of Bioinformatics in LatAm, indicating data on the number of publications by countries, the topics of these publications, and the sources of their funding. In addition, he highlighted that many countries such as Venezuela and Ecuador are still in the first steps of their bioinformatics development. Afterward, there was an open discussion of the weaknesses and strengths of LatAm countries to develop Bioinformatics. Dr. Hugo Naya emphasized that the main objective on which we have to focus is to encourage cooperation, employing accessible training, and software development, for which it is not necessary to have a lot of technological capacity and other resources. Then, Dr. Francisco Flores explained that there is little scientific production of Bioinformatics in Ecuador, and educational and collaboration opportunities for Bioinformatics in this country are also scarce. Additionally, he commented on the concern for maintaining the acquired high-performance computing machines and other equipment, once the effort is made to acquire them. Later, Dr. Ascanio Rojas mentioned the importance of breaking the English language barrier and getting to know our neighbors as potential collaborators. In the discussion panel, all guests agreed that understanding bioinformatics data correctly is more important than having powerful computational infrastructure. The RSG organizers assumed the responsibility of creating a platform where each member can put their available computing capacity at the service of others, as well as the projects in which they are working, which could promote future cooperation. Figure 5 shows the graphical summary of the round table discussion.

## Award winners

The 1SEVJIB selected two winners for the best oral and poster presentations. The winners were chosen by the organizing committee using an evaluation rubric of the quality of each presentation.

The best oral presentation was for Viviam Bermúdez from the Norwegian University of Science and Technology with the presentation titled “ *Boolean Logic Modeling of Macrophage Polarization*”. This research focused on understanding the role of entities such as eicosanoids and enzymes on the regulatory factors that drive macrophage polarization, this research aimed to assemble a Boolean logic model of macrophage polarization using a systems biology approach. A previous model of macrophage differentiation was used and through the evaluation of the literature, it was extended with 38 entities and 82 key interactions in polarization. They tested the model under 23 different perturbations and experimental conditions and it was able to correctly predict 15 expression profiles, where one of these was validated experimentally. This model provides a tool to improve the study of eicosanoids in macrophage polarization, which is suitable for designing future experiments and new drugs.

The best poster presentation was awarded to Mario Jurado from the Universidad Santo Tomás (Colombia) with a poster titled “ *Stability of protein-ligand complexes between glucosyltransferase and citronellal terpenes*”. This research focused on finding new alternatives for the control of dental caries using Molecular Dynamics simulations. According to this research, some compounds derived from essential oils of *Cymbopogon martinii*, *Cymbopogon nardus,* and *Thymus vulgaris* are promising drugs against *Streptococcus mutans,* the main pathogen of dental caries.

## Conclusions

The 1SEVJIB represents the first joint effort between two Latin American RSGs to organize a common event. As a result, this first bi-national conference, focused on Bioinformatics and Computational Biology in Ecuador and Venezuela, was successfully carried out. This experience showed the scientific quality, skills, and motivations of young Bioinformatics researchers from Ecuador, Venezuela, and other LatAm countries.

Moreover, the 1SEVJIB showed the need to create spaces for the promotion of Bioinformatics in our countries, where the new generation of researchers have the possibility of demonstrating innovative work, in their native language, on a global stage. As the event was conducted in Spanish, our participants had the opportunity to build important skills to start their careers in Bioinformatics, despite the potential language barriers. This aspect is important for Spanish speaking countries, especially those from LatAm, where the English proficiency level, as measured by the “English First, English Proficiency Index” in 2016, ^15^ showed that the 18-20 years age group falls behind the world average by 3.8 points. Our 1SEVJIB fuelled the organization of similar, bigger initiatives like the first Symposium of Spanish-Speaking Students of Bioinformatics and Computational Biology (SEH ^2^-Bioinfo, https://seh2bioinfo.netlify.app/), organized by 9 RSGs from spanish-speaking countries in LatAm and Spain, including the RSG Ecuador and RSG Venezuela.

In addition, this symposium promoted the creation of communication and cooperation networks between LatAm researchers who can join forces to produce scientific knowledge in terms of peer-reviewed articles, thesis projects, internships, among other activities related to Bioinformatics.

The popularization of virtual events, such as the one described in this document, has been the common denominator in the scientific community since the COVID-19 pandemic started. These activities have been accelerated and promoted because they overcome the geographical, administrative, and economic barriers that on-site events entail. Thanks to these conditions and the support of the ISCB-SC, we organized the 1SEVJIB, a platform where students and young researchers interested in Bioinformatics shared their knowledge, learned from LatAm leading experts, and discussed the opportunities and challenges of this field in our countries and region. Furthermore, collaboration and joint work were strengthened, in order to ensure the continuity and promotion of the Bioinformatics and RSGs in LatAm.
